# Quality of life and salivary output in patients with head-and-neck cancer five years after radiotherapy

**DOI:** 10.1186/1748-717X-2-3

**Published:** 2007-01-05

**Authors:** Pètra M Braam, Judith M Roesink, Cornelis PJ Raaijmakers, Wim B Busschers, Chris HJ Terhaard

**Affiliations:** 1 Department of Radiotherapy, University Medical Center Utrecht, Utrecht, The Netherlands; 2 Department of Biostatistics, Utrecht University, Utrecht, The Netherlands

## Abstract

**Background:**

To describe long-term changes in time of quality of life (QOL) and the relation with parotid salivary output in patients with head-and-neck cancer treated with radiotherapy.

**Methods:**

Forty-four patients completed the EORTC-QLQ-C30(+3) and the EORTC-QLQ-H&N35 questionnaires before treatment, 6 weeks, 6 months, 12 months, and at least 3.5 years after treatment. At the same time points, stimulated bilateral parotid flow rates were measured.

**Results:**

There was a deterioration of most QOL items after radiotherapy compared with baseline, with gradual improvement during 5 years follow-up. The specific xerostomia-related items showed improvement in time, but did not return to baseline. Global QOL did not alter significantly in time, although 41% of patients complained of moderate or severe xerostomia at 5 years follow-up. Five years after radiotherapy the mean cumulated parotid flow ratio returned to baseline but 20% of patients had a flow ratio <25%. The change in time of xerostomia was significantly related with the change in flow ratio (p = 0.01).

**Conclusion:**

Most of the xerostomia-related QOL scores improved in time after radiotherapy without altering the global QOL, which remained high. The recovery of the dry mouth feeling was significantly correlated with the recovery in parotid flow ratio.

## Background

Patients with head-and-neck cancer have to cope with many aspects of their life-threatening disease. They have to deal with the diagnosis and the treatment as well as with the impact on physical, psychological and social functioning. Radiotherapy (RT) is a treatment modality, sometimes combined with surgery that can give considerable acute and long-term side effects to the oral cavity. One of the effects is a dry mouth (xerostomia), due to irradiation of the salivary glands. Furthermore, chewing and swallowing difficulties, impaired taste or an increased incidence of dental caries or oral candidiasis can occur [1,2].

Quality of life (QOL) questionnaires have been utilized for several years in the follow-up of patients with head-and-neck cancer, and impaired QOL has been reported until years after RT [3,4]. Up to 12 months after RT the xerostomia-related QOL scores follow the general pattern of salivary flow rates [5,6]. The long-term relationship between the individual's perception of a dry mouth, the QOL and the objective parotid salivary output however, has not been determined.

We performed a prospective study in patients with head-and-neck cancer receiving RT. The first aim of the study was to assess the long-term change in time of the QOL. The second aim was to investigate the relationship between change in time of the subjective outcome and the objective parotid flow measurements. We also analyzed the relationship between the change in time of the subjective outcome and the mean parotid dose (D_par_), and the mean submandibular dose (D_subm_). Earlier we presented the short-term and long-term parotid flow data of this study group [7,8]. In this paper, we present results after a minimum follow-up of 3.5 years.

## Methods

### Patients

From July 1996 till October 1998, patients with head-and-neck cancer that received primary or postoperative RT with curative intent were included in the study. Other inclusion criteria were no previous RT or surgery of the parotid glands, no history of suffering from malignancies or other diseases of the parotid glands and WHO 0–1. Patients with evidence of (p)N2c-N3 (TNM staging system 1997) or distant metastases, were excluded. All patients treated with induction or concomitant chemotherapy were excluded, because this might influence the parotid function [9]. No patient used medication known to affect the function of the salivary glands.

**Figure 1:**
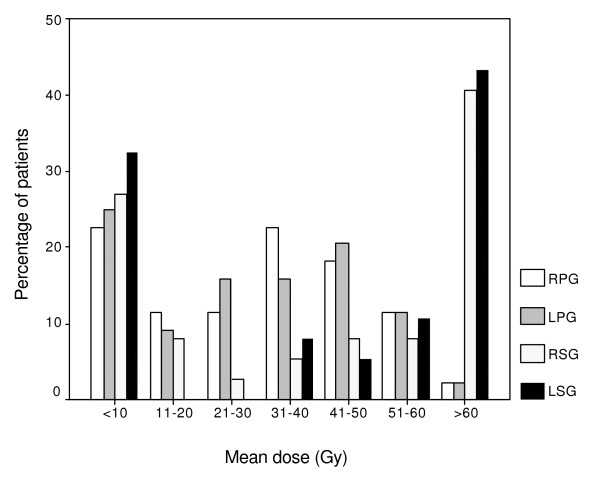
Distribution of the mean dose (Gy) of the different glands presented as the percentage of patients. *Abbreviations*: RPG = right parotid gland; LPG = left parotid gland; RSG = right submandibular gland; LSG = left submandibular gland.

One hundred and eight patients met the inclusion criteria. At minimum follow-up of 3.5 years (hereafter referred to as 5-years follow-up), 27 died, 6 were too ill to participate, 3 had surgery for recurrence, 7 refused participation, 12 had incomplete data and 9 were lost to follow-up. This resulted in 44 patients who were able to fill in the questionnaire and could be assessed (table 1). Only data received from these 44 patients were analyzed. Patients were treated predominantly with 6-MV X-rays from a linear accelerator using parallel-opposed lateral beams. The irradiation varied with the diagnosis, according to generally accepted treatment strategies. The mean dose prescribed to the primary target was 61.1 Gy, ranging from 40 to 70 Gy. The right D_par _was 28.3 Gy (range 1–62 Gy) and the left D_par _was 27.9 Gy (range 0–62 Gy). The right D_subm _was 39.9 Gy (range 1–71 Gy) and the left D_subm _was 41.0 Gy (range 0–70 Gy). The distribution of the mean doses of the different glands is presented in figure 1. Due to the different tumor sites with 43% laryngeal cancer, these relatively low doses to the parotid glands were obtained.

**Table 1:** Patient and tumor characteristics (*n *= 44)

Mean age (range)	56	(24–78) y
Gender		
Female	10	(23%)
Male	34	(77%)
Mean follow-up time (range)	56	(44–72) months
since end of radiotherapy		
Tumor site		
Larynx	19	(43%)
Floor of mouth/oral cavity	7	(16%)
Oropharynx	4	(9%)
Nose (nasal cavity)	4	(9%)
Hypopharynx	1	(2%)
Nasopharynx	1	(2%)
Other	8	(18%)
Surgery preradiotherapy		
Local	6	(14%)
Local + regional	11	(25%)
No	27	(61%)
Stage (TNM staging system 1997)		
T1	7	(22%)
T2	16	(50%)
T3	5	(16%)
T4	4	(12%)
Not applicable/recurrent	12	
N0	27	(84%)
N1	4	(13%)
N2b	1	(3%)
Not applicable/recurrent	12	

### Questionnaire

Patients completed a questionnaire before treatment and 6 weeks, 6 months, 12 months, and at least 3.5 years (mean 56 months, range 44–72 months) after treatment. The questionnaire consisted of the EORTC QLQ-C30(+3) and QLQ-H&N35.

The EORTC QLQ-C30 is a widely used questionnaire and contains QOL issues relevant to a broad range of cancer patients. It includes five functional scales, three symptom scales, a global QOL scale and six single items [10]. Version 30(+3) contains two additional items on role functioning and one additional item on overall health. The EORTC QLQ-C30(+3) is meant to be used in conjunction with a tumor specific module.

The EORTC QLQ-H&N35 is a module used for the assessment of health-related QOL in patients with head-and-neck cancer [11]. It contains seven symptom scales and six symptom items. It is designed to be used together with the core QLQ-C30 and has been validated in 622 head-and-neck cancer patients from 12 countries [12].

After transformation all items and scales range in score from 0 to 100. High scores for a functional or global QOL scale represent good functioning, or a high QOL, whereas a high score for a symptom scale or single item represents a high level of symptomatology or problems [10].

### Saliva collection

Parotid flow rates were measured at the same time points as the QOL measurements. No oral stimulus was permitted for 60 min before saliva collection. Stimulated parotid saliva was simultaneously collected separately from left and right parotid gland using Lashley cups. These cups were placed over the orifice of the Stenson's duct. Stimulation was achieved by applying three drops of a 5% acid solution to the mobile part of the tongue every 30 seconds and collection was carried out for 10 min. The volume of the saliva was measured in tubes by weight. It was assumed that the density of the parotid saliva was 1 g/ml. The flow rate was expressed for each separate gland in milliliters per minute (ml/min). The left and right parotid flow rates were added together and converted into the percentage of baseline flow rates (flow ratio). A complication was defined as cumulated stimulated parotid flow rate of <25% of the pre-RT flow rate.

### Statistics

**Figure 2:**
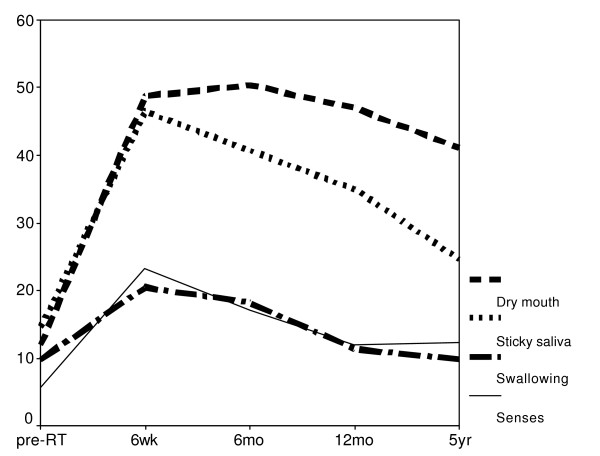
Mean scores over time of the single items dry mouth, sticky saliva, swallowing and senses (QOL-H&N35). High scores imply a high level of symptoms.

**Figure 3:**
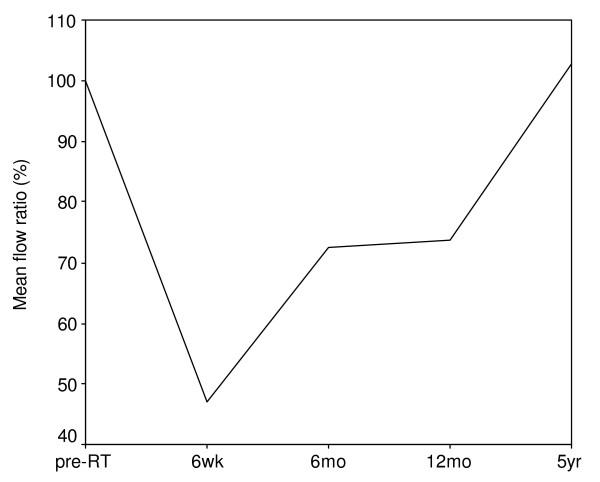
Stimulated parotid flow rates (mean value) at different timings after radiotherapy. Time 0 means before radiotherapy. The cumulated flow rates are expressed as the percentage of the pre-radiotherapy flow rates. Note: the x-axis is non-linear.

The data of all items and scales of the EORTC QLQ-C30(+3) and the EORTC QLQ-H&N35 were transformed to a 0–100 scale for presentation according to the guidelines of the EORTC (table 2, figure 2, figure 3). For the analysis we decided to use the non-transformed data, because of the discrete and ordinal characteristics of the response. Missing data were excluded from analyses. Mixed effects ordinal regression techniques were used to account for dependency between observations in time and to examine relationships between the response of interest and possible explanatory variables time, D_par_, D_subm _and parotid flow ratio. Dr Hedekers software package Mixor was used to obtain estimates of the model parameters.

**Table 2:** Mean scores of the scales and single items of questionnaire for patients with cancer of the head- and-neck treated with radiotherapy with or without surgery. A significant outcome presents a significant change in time towards improvement starting 6 weeks after RT.

	pre-RT	6 weeks	6 mo	12 mo	5 years	Significance
**EORTC QLQ-C30(+3)**						
Functioning scales*						
Cognitive	90.1	88.0	88.6	90.2	87.3	NS
Emotional	75.8	83.5	83.2	85.5	83.7	NS
Physical	80.6	85.0	85.0	87.0	85.1	NS
Role	75.8	83.5	83.2	85.5	83.7	NS
Social	86.9	88.8	89.4	93.6	87.8	NS
Global QOL*	71.6	73.3	80.1	81.6	80.6	NS
Symptom scales†						
Fatique	24.3	30.5	26.8	23.4	27.5	p < 0.01
Pain	14.3	11.6	15.0	8.6	12.0	NS
Nausea and vomiting	3.6	7.4	1.2	2.2	0.8	p < 0.01
Single items†						
Dyspnoea	16.7	13.2	18.7	15.4	14.3	NS
Insomnia	24.6	25.6	21.1	17.0	15.5	p < 0.01
Appetite loss	7.9	14.0	8.9	7.7	10.1	p < 0.05
Constipation	3.2	10.1	5.7	7.7	7.0	NS
Diarrhoea	1.6	2.3	1.6	6.0	0.0	NS
Financial problems	5.6	5.4	4.1	5.1	5.7	NS
**EORTC QLQ-H&N35**						
Symptom scales-single items†						
Pain	10.6	19.4	19.1	15.5	9.5	p < 0.01
Swallowing	9.8	20.5	18.2	11.4	9.9	p < 0.01
Senses (taste/smell)	5.6	23.3	17.1	12.0	12.3	p < 0.01
Speech	23.8	17.8	15.0	11.5	14.4	p < 0.01
Social eating	7.9	19.8	14.8	10.7	10.6	p < 0.01
Social contact	4.0	6.2	2.6	3.8	4.6	NS
Sexuality	14.8	78.7	17.1	20.7	25.4	NS
Teeth	10.5	31.8	21.1	19.8	18.7	NS
Open mouth (trismus)	11.1	14.0	15.5	9.4	13.9	NS
Dry mouth	11.9	48.8	50.4	47.0	41.1	p = 0.01
Sticky saliva	14.6	46.5	40.7	35.0	24.6	p < 0.01
Cough	17.5	23.3	26.0	18.8	13.5	p < 0.01
Nutrition supplements	7.3	32.6	12.2	12.8	4.9	p < 0.01

*Higher score indicates better function. † Higher score indicates more symptoms. ‡ Significance based on ordinal regression model using non-transformed data. QLQ, quality of life; RT, radiotherapy; NS, not significant.

## Results

### QOL

A deterioration of almost all scales and items in QLQ-H&N35 was noted after RT and generally no effect was seen in the QLQ-C30(+3) questionnaire (table 2). Most items improved in time but not all reached baseline values (figure 2). The specific xerostomia related items dry mouth and sticky saliva showed deterioration 6 weeks after RT, which continued for dry mouth till 6 months. Thereafter both items showed an improvement but at 5 years after RT their values remained higher than baseline. We investigated the relation between the change in time of the various parameters starting after RT and not the relation at specific time points. At 12 months follow-up, 49% of the patients complained of a moderate or severe dry mouth, which slightly improved to 41% of the patients at 5 years. The functional scales of the QLQ-C30(+3) showed no significant alteration after RT. The mean scores before RT were already relatively high and showed only slight differences in time, but no significant change caused by RT. The global QOL was also not significantly altered in time in spite of the remaining dry mouth complaints.

### Parotid flow measurements

Parotid flow rate diminished immediately after RT with a maximal deterioration at 6 weeks, and increased progressively in time. The mean stimulated parotid flow rate was 0.29 (SD 0.19) ml/min before RT. Six weeks after RT the mean stimulated parotid flow rate decreased to 0.14 (SD 0.08) ml/min, with thereafter an increase to 0.19 (SD 0.13) ml/min, 0.19 (SD 0.13) ml/min and 0.26 (SD 0.17) ml/min, respectively 6 months, 12 months and 5 years after RT. Figure 3 shows the mean parotid flow ratio at the different measurement time points. Because of the variability in flow rates, the flow ratio can reach percentages above 100%. The respective median parotid flow ratios were 35%, 47%, 69%, and 79% for 6 weeks, 6 months, 12 months, and 5 years. The percentage of patients with a complication declined from 46% at 6 weeks after RT to 20% at 5 years after RT (table 3).

**Table 3:** Percentage of patients divided into three groups by the flow ratio at different time points (n = 44).

	6 weeks	6 mo	12 mo	5 years
Flow ratio				
<25%	46	35	24	20
25%–<75%	28	30	35	24
75%	26	35	41	56

### Relationship between subjective and objective parameters

#### Global QOL, dry mouth, sticky saliva and flow ratio

We investigated the relationship between the change in time of the subjective outcome of the questionnaire and the change in time of the objective stimulated parotid flow ratio. As objective explanatory variable we used the sum of the left and right parotid flow ratio. No significant relation was found between the change in global QOL and the change in flow ratio (*p *= 0.60). A significant relation between the flow ratio and dry mouth was found (*p *= 0.01). We found no evidence that the reduction of problems with sticky saliva could be explained by parotid flow (*p *= 0.79), adjusting for time revealed a significant time effect (*p *= 0.003). In other words, the improvement of problems with sticky saliva could be explained by time and was not due to the improvement of the parotid flow.

#### Global QOL, dry mouth, sticky saliva and mean dose

No clear relation was found between the change in time of the dry mouth item and D_par _or D_subm_. We found no significant relation between the change in time of the global QOL or sticky saliva and the mean dose to the various salivary glands. We also did not find a combined relationship.

## Discussion

This is the first long-term prospective study of the QOL combined with parotid salivary output of patients with head-and-neck malignancies treated with RT. We found a deterioration of most of the QOL items after completion of radiotherapy compared with baseline, with improvement during 5 years follow-up, even after 12 months. The specific xerostomia-related items improved, but did not return to baseline. Global QOL did not alter significantly in time, despite the fact that 41% of patients complained of a dry mouth at 5 years follow-up. Similar to the partial recovery of the dry mouth, the stimulated parotid flow rates gradually improved after radiotherapy, even after 12 months. We have presented this recovery in more detail previously [7]. This improvement of the dry mouth was significantly related with the improvement of the parotid flow ratio (*p *= 0.01).

The finding of a moderate to severe dry mouth years after treatment and a normalized quality of life is consistent with other studies [4,13-16]. It might be explained by adaptation of the patients to their disabilities, as I quote a patient: "doctor, I feel fine and I do not have a dry mouth" after which he took a sip of water out of a bottle he carried with him. It is known that the QOL varies according to gender and age and that gender and age have to be taken into consideration for analyses [17]. But because of the relatively small number of patients in the present study, differentiation between men and women and age could not be studied. It should be remarked that at baseline most patients were preoperative with the tumor still in situ or just post-operative. Both situations may affect the QOL and related parameters and improvement in time. As all patients had this baseline situation, the analyses should be viewed in this perspective.

This study population consisted of 44 survivors derived from a larger group of patients. We only analyzed the group of surviving patients knowing that this is a favourable group and not representative of an average population. Analyses between survivors and non-survivors have been reported previously, and showed statistical difference between the flow ratio in favour of the survivors, but only at 6 weeks and 6 months and not at 12 months [7]. This report shows that in patients who do survive, improvement over time can be seen.

There are various ways of recording parotid gland toxicity. Several head-and-neck cancer specific QOL questionnaires have been conducted and validated for subjective measurement [10-12,18,19]. We used the EORTC-QLQ-C30(+3) and the EORTC-H&N35 questionnaires which are well-validated and widely used. For objective methods salivary flow measurement using sialometry or scintigraphy have been reported [20-23]. The most adequate parameter to evaluate the function of the parotid gland is objective stimulated parotid flow measurement and consequently we used this method [24]. Recently MRI, SPECT, and PET have been used to quantify the parotid gland radiation response, but they still have to prove their value [25-28].

**Figure 4:**
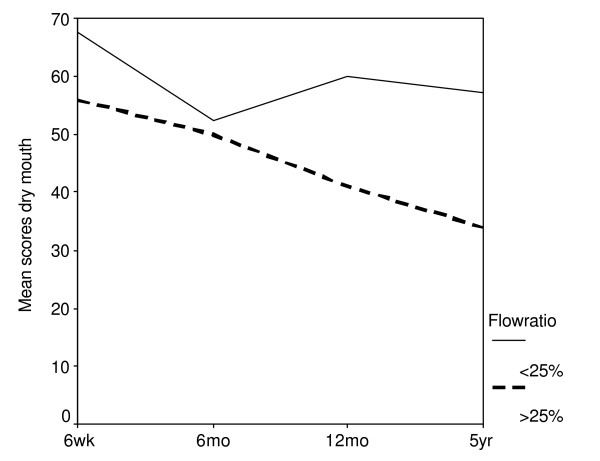
Mean scores over time of the single item dry mouth (QOL-H&N35). High scores imply a high level of symptoms. A division has been made between patients with and without a complication, defined as stimulated cumulated parotid flow rate <25% of the pre-radiotherapy flow rate.

Several institutions have reported on subjective QOL and xerostomia in relation with salivary flow rates in the short-term with analysis at fixed time points. Henson et al found that the xerostomia-related QOL scores followed the general pattern of parotid flow rates, till 1-year follow-up [6]. Parliament et al reported an inverse correlation between the unstimulated and stimulated whole salivary flow and xerostomia-specific items at one month, which disappeared three months and twelve months after treatment [29]. Blanco et al found a strong correlation between the stimulated salivary function and the QOL scores 6 months after RT and a nonsignificant trend towards improvement in the mean QOL scores between 6 and 12 months [5]. In our long-term analysis in which we focused on changes in time and not at relations at fixed time points, a significant correlation was found between the flow ratio recovery and the changes in the dry mouth item (*p *= 0.01). Previously we found a significant association between time and flow ratio [7]. Five years after RT the mean parotid flow ratio returned to baseline while 41% of patients still experienced a moderate to severe dry mouth. A possible explanation is that patients who had a flow ratio <25% complained the most of a dry mouth. A flow ratio <25% appeared to be the best definition for objective parotid gland toxicity [24]. The number of this group of patients diminished in time, constituting almost one-fifth of the total at 5 years. The number of patients with a flow ratio between 25% and 75%, became smaller and the number of patients with a flow ratio >75% (and exceeding 100%) became larger in time (table 3). In subanalyses we made a division between patients with and without a complication (flow ratio <25%, as defined earlier). A difference between the two groups in time was seen. At all the time points, patients with a complication had higher score results (more complains) but this was not statistically significant (figure 4). The low number of patients in the two groups combined with the large number of possible answers (4) may obscure the difference between the two groups. Further research using a larger group of patients is required. Another explanation is that not only the parotid glands are responsible for the dry mouth feeling. There might be an influence of the submandibular glands and/or the minor salivary glands of the palate. In our analysis neither the D_par _nor the D_subm _was conclusively associated with the xerostomia-specific items. This is in agreement with others who looked at fixed time points [30]. We also did not find a combined influence of the D_par _and the D_subm_. As can be seen in figure 1, the D_subm _was not normally distributed. Most patients either received a very low or a very high dose. This can contribute to the negative outcome. Eisbruch et al found a significant correlation between the mean dose to the oral cavity and the xerostomia scores at different time points [18]. In their report, the oral cavity mean dose represented the RT effect on the minor salivary glands. This indicates that it may be beneficial to spare the noninvolved oral cavity to further reduce xerostomia. In the contrary Jellema et al showed no significant association between xerostomia and the oral cavity mean dose [30]. As there is till now to our knowledge, unfortunately, no conclusive relation, the oral cavity mean dose is not used at our institute.

## Conclusion

Xerostomia-related QOL improved in time after radiotherapy without accompanying changes in global QOL. The global QOL remained high during time and no statistically significant changes were observed. The recovery of the dry mouth feeling was significantly related with the change in parotid flow ratio. Although the parotid flow rates recovered till baseline at 5 years follow-up, 41% of the patients complained of a moderate to severe dry mouth.

## Competing interests

The author(s) declare that they have no competing interests.

## Authors' contributions

PB participated in the design of the study, carried out the subjective and objective measurements at the different time points, performed statistical analyses, and drafted the manuscript. JR participated in the design of the study, carried out the subjective and objective measurements at the different time points and revised the manuscript critically. CR made substantial contribution to conception of the study and revised the manuscript critically. WB made the analysis and interpretation of the data, and has been involved in drafting the manuscript. CT participated in the design of the study, contributed to the acquisition of data and revised the manuscript critically. All authors read and approved the final manuscript.
